# Modern Detection of Prostate Cancer's Bone Metastasis: Is the Bone Scan Era Over?

**DOI:** 10.1155/2012/893193

**Published:** 2011-10-16

**Authors:** Bertrand Tombal, Frederic Lecouvet

**Affiliations:** ^1^Division of Urology, Cliniques Universitaires Saint Luc, Université Catholique de Louvain, 1200 Brussels, Belgium; ^2^Division of Radiology, Cliniques Universitaires Saint Luc, Université Catholique de Louvain, 1200 Brussels, Belgium

## Abstract

Prostate cancer cells have an exquisite tropism for bone, which clinically translates into the highest rate of bone metastases amongst male cancers. Although in the latest years there has been an active development of new “bone targeted” therapies, modern diagnostic techniques for bone metastases still relies mostly on ^99m^Tc bone scanning (BS) and plain X-ray. BS dramatically lacks specificity and sensitivity. Recent publications using modern imaging technologies have clearly pinpointed that BS grossly underestimates the true prevalence of bone metastasis. In addition BS does not allow tumour measurement and is, therefore, not appropriate to monitor response to therapy. This might be extremely important in patients harbouring high-risk localized disease that are eventually candidate for local therapy. Here we reviewed what are the emerging imaging strategies that are likely to supplant BS and to what extent they can be used in the clinic already.

## 1. Introduction

In men over the age of 50, prostate cancer (PCa) is the most commonly diagnosed cancer and the second leading cause of death by cancer [1]. With the intense use of PSA testing, most PCa are diagnosed at an early stage, and most are candidate to intent-to-cure therapies such as radical prostatectomy, external beam radiation therapy, or seeds implant. Initially essentially intended for low- or intermediate-risk disease, local therapies are now more often indicated in patients with high-risk localized disease and locally advanced disease. Indeed, hormone therapy has failed to demonstrate to increase overall survival when it was not associated with a local treatment [2]. In that high-risk population; however, it is critical to precisely rule out the presence of metastases since as for today it still represents the tipping point for excluding local control. 

PCa cells spreading out of the prostate show an exquisite tropism for bone. In most patients, the initial seeding of metastatic deposits occurs in the hematopoietic red marrow of the axial skeleton leading to the formation of bone metastases (BMs). BMs represent the initial and the main metastatic site in about 80% of PCa patients, therefore, being one of the most important prognostic factors [3, 4]. Skeletal complications, most commonly designed as “skeletal-related events (SREs),” account for most of the PCa's morbidity and mortality [5]. Replacement of hematopoietic tissues in the bone marrow by PCa cells leads to anaemia while abnormal tissue growing in the bone marrow can lead to pain, fractures, and spinal cord compression. As for today, despite the development of bone-targeted strategies, BMs are still considered incurable [6]. 

The main phenotypic manifestations of PCa BMs are their tropism to the axial skeleton (skull, vertebra, ribs and collar bone, scapula, and proximal femur) and their most often osteoblastic appearance, resulting from the stimulation of osteoblasts [6]. BM, are often associated with increased levels of serum markers of osteoblastic proliferation, such as bone-specific alkaline phosphatase [7]. 

Interestingly, there has been no major evolution in the diagnostic algorithm for BM in the recent years. Most international guidelines still recognized ^99m^Tc bone scintigraphy (BS) and plain X-ray radiography as the cornerstone diagnostic techniques to detect and follow BM [8, 9]. Most modern clinical trials still incorporate BS as a major component to define time to progression, although it is recognized as not appropriate to measure tumour response and required serial examinations to define progression [10–12]. There are, however, several technological developments addressing this important diagnostic aspect. Here we will review some of these developments.

## 2. Why and When Bone Imaging Is Required?

Early BM detection is critical in the management of patients with high-risk PCa. Newly diagnosed patients with localized disease and no metastases may benefit from radical treatment with curative intent. In contrast, most guidelines recognize that patients with BM should be kept away from local therapy to avoid unnecessary side effects and treated with systemic therapy [9]. With modern PSA-based diagnostic strategies, many patients are diagnosed while they are still asymptomatic. In screening trials, BMs are detected at diagnosis in less than 10% of the patients [13]. This means that there is no need to perform an initial BS in every new patient. PSA value and Gleason's score at diagnostic remain the strongest BM's indicators. In a study conducted on 60 patients with newly diagnosed PCa, Rana et al. demonstrated that the positive predictive value of a PSA >100 ng/mL was 100% [14]. Together with PSA, cT_3-4_ stage and a Gleason score >7 are the other predictors of BM; their positive predictive value being, respectively, 71.4% and 81% [14]. Based on this trial and others, the EAU guidelines recommend that […] *a staging bone scan may be superfluous if the serum PSA concentration is less than 20* *ng/mL in asymptomatic patients with well-, or moderately differentiated tumours. In contrast, in patients with poorly differentiated tumours and locally advanced disease, a staging bone scan should be obtained irrespective of the serum PSA value* […] [9]. Briganti et al. have developed a risk stratification tool to select patients requiring initial imaging from a series of 853 consecutive patients [15]. Their classification and regression tree (CART) stratifies patients into low risk (biopsy Gleason ≤7, cT_1-3_, and PSA <10 ng/mL), intermediate risk (biopsy Gleason ≤7, cT_2-3_, and PSA >10 ng/mL), and high risk (biopsy Gleason >7) conferring a risk of BM of 1.8%, 8.5%, and 16.4%. Briganti's regression tree shows higher sensitivity (87.5%) compared to the EAU, AUA, and NCCN guidelines [8, 9, 16].

Later in the course of the disease, BM detection may be discussed in case of PSA recurrence after radical treatment or when the tumour becomes resistant to castration (CRCP). This information may be important to guide initiation of hormone therapy, chemotherapy, or bone-targeted agents. Gomez et al. have evaluated the use of BS in 153 patients presenting with a PSA recurrence after radical prostatectomy. This study demonstrated that it is unlikely to have a positive BS in patients with a serum PSA of <7 ng/mL except in case of skeletal symptoms [17]. Pound et al. have reviewed a large series of radical prostatectomy performed at the Johns Hopkins University and identified 304 patients with a subsequent PSA rise [18]. The median actuarial time to metastases was 8 years from the time of PSA level elevation. In survival analysis, time to biochemical progression ≤2 years (*P* < 0.001), Gleason's score ≤8 (*P* < 0.001), and PSA doubling time ≤10 months (PSADT) (*P* < 0.001) were predictive of the probability and time to the development of metastatic disease. Choueiri et al. have reviewed case notes of 292 patients from CaPSURE who had recurrence and had undergone at least 1 imaging study (BS, computerized tomography (CT), or magnetic resonance imaging (MRI) of the abdomen and the pelvis) [19]. Overall only 11% patients showed a positive imaging study, and this was unlikely to occur when PSA was ≤5 ng/mL or PSADT ≥10 months [19]. 

Smith et al. have reported the natural history of 201 non-metastatic (M0) CRCP patients consisting of the placebo group of an aborted trial with zoledronic acid [20]. At 2 years, only 33% of patients had developed BM. A baseline PSA level >10 ng/mL and a high PSA velocity independently predicted shorter time to first BM. Patients with a PSADT <6 months were those most likely to rapidly develop BM. More recently, Smith et al. have reported the natural history of placebo group of another M0 CRCP study conducted with the endothelin receptor A inhibitor, atrasentan [21]. Patients included in the atrasentan trial had a shorter PSADT (mean ± SD: 5.9 ± 3.62 months) than those in the zoledronic acid trial (mean ± SD: 9.7 ± 4.7 months) [20, 21]. In multivariate analyses, a baseline PSA ≥13.1 ng/mL was associated with shorter time to the first BM and BM-free survival.

In conclusion, all these data pinpoint at a high Gleason's score, a high PSA, and a short PSADT being important to decide which patients should receive bone investigation to search for BM. Taken together, these data should help the clinician schedule follow-up examination. 

### 2.1. ^99m^Technetium (^99m^Tc) Bone Scintigraphy

Today, standard diagnostic algorithms of bone metastases still rely primarily on ^99m^Tc methylene diphosphonate (MDP) bone scintigraphy (BS). BS has been used for decades as the first-line modality for the screening of PCa bone metastases [22, 23]. ^99m^Tc-MDP is a nonspecific marker of osteoblastic activity. Studies using microautoradiography have demonstrated that ^99m^Tc-MDP localizes along mineralization fronts. The isotope is occasionally found in the substance of the osteoid but is absent from the cytoplasm and nuclei of osteoblasts and osteocytes [24]. ^99m^Tc-MDP accumulates in response not only to tumour but also to degenerative joint disease, benign fractures, and inflammation [25–27]. Therefore, BS detects bone metastases at an advanced stage of tumour infiltration, when osteoblastic reaction to metastatic cell deposit has occurred [28]. Sensitivities reported in the literature range between 62 and 89% and, therefore, could be considered acceptable [28]. BS's main problem is its low specificity so that its diagnostic effectiveness has been widely questioned in the literature [29, 30]. Indeed, in many cases, regions of increased uptake cannot be definitively characterized negative or positive for malignancy. Routinely, it will end up in reading characterized as “equivocal,” “possible,” “suspicious,” “likely,” “highly suspicious,” “almost certain,” a series of definition encompassing all cases in which imaging findings could not be categorized confidently as metastatic or benign, regardless of the level of incertitude. Usually equivocal BS uptakes will be characterized by targeted X-ray to distinguish benign (fracture, Paget's, degenerative joint disease, etc.) from malignant (metastatic) origin [31]. This association is imperfect, and the diagnosis may remain equivocal after this workup. In clinical practice, a normal bone radiograph associated with an abnormal scan is highly suggestive of BM [32]. But this conclusion invokes conventional wisdom or suggestion more than it relies on a robust approach. Conventional wisdom may be acceptable in clinical practice to assess a fractures risk; it becomes questionable when it comes to definitive decisions in oncology [33].

### 2.2. SPECT and SPECT CT

Noteworthy, guidelines do not provide technical recommendations for BS. In many centres BS is limited to anterior and posterior planar images. Standard planar BS can be improved by single-photon emission computerized tomography (SPECT) on selected areas such as the lower thoracic and lumbar spine region or even on the entire axial skeleton (Whole-body SPECT) [33–35]. In cancer patients in general and in PCa in particular, SPECT enhances both sensitivity and specificity for the detection of BM [34–36]. Even-Sapir et al. have compared BM detection by BS, SPECT, ^18^F-Fluoride PET, and ^18^F-Fluoride PET/CT in 44 patients with high-risk PCa, including 23 (52%) with BM [36]. The sensitivity, specificity, positive predictive value (PPV), and negative predictive value (NPV) of planar BS were 70%, 57%, 64%, and 55%, respectively, and of SPECT were 92%, 82%, 86%, and 90%, respectively. SPECT identified 62% of the lesions overlooked by planar BS. The major advantage of SPECT is that the added benefit of tomographic imaging required few extra radiation dose and only slight added cost, since most modern cameras now offer the possibility of whole-body SPECT [33]. Nozaki et al. have assessed the value of SPECT in the detection of PCa BM in the lumbar vertebrae in 39 patients, all of them having increased ^99m^Tc uptake on BS [37]. Definitive diagnosis of BM was established by MRI. Sensitivity, specificity, PPV, and NPV of bone SPECT were 95.9%, 73.1%, 72.3%, and 96.1%, respectively. Helyar et al. have investigated the additional value of SPECT/CT over Tc-99m BS and SPECT on 50 lesions detected by BS in 40 patients [38]. On reporting the planar study and SPECT scans, reviewers rated 61% of lesions as equivocal. On reporting the SPECT/CT scans, only 8% of lesions were rated as equivocal, 24% were rated as malignant, and 68% as benign.

As a conclusion, SPECT and SPECT/CT provide better accuracy than Tc99m BS in differential diagnosis of lumbar BM. Because whole-body SPECT is time-consuming and not widely available, it is not yet recognized as the “optimal state-of-the art” screening technique, although it is available on most standard BS machine and can be performed at minimal cost increased.

### 2.3. Metabolic Imaging, PET, and PET/CT

Tumour detection, using positron emission tomography (PET) and PET/CT, has emerged as a standard imaging techniques in oncology, since PET/CT has demonstrated higher sensitivity for the early detection of metastases, including BM, in various malignancies [39–41]. Unfortunately, the most widely used metabolic marker, ^18^F-FDG PET, has little or even no interest in PCa patients. Ghanem et al. have demonstrated that ^18^F-FDG-PET alone or using PET-CT image fusion, is less sensitive than MRI in the detection of bone metastases [42]. ^18^F-(Sodium) Fluoride is a high affinity bone-seeking agent usually considered as a promising substitute for ^99m^Tc-MDP. In the aforementioned series of 44 high-risk PCa, Even-Sapir et al. has reported sensitivity, specificity, PPV, and NPV of, respectively, 100%, 62%, 74%, and 100% for ^18^F-Fluoride PET and of 100% for all parameters for ^18^F-Fluoride PET/CT [36]. Of the 156 bone lesions detected by ^18^F-Fluoride, 81 lesions (52%), including 34 metastases, were overlooked with normal appearance on planar BS. In contrast, the other aforementioned trial by Ghanem et al. failed to show superiority of ^18^F-Fluoride PET over MRI in BM detection [42]. 

Future studies will focus on newer markers such as ^11^C- or ^18^F-labeled choline and acetate, ^11^C-methionine, and ^18^F-fluorodihydrotestosterone [26, 36, 40–44]. Kotzerke et al. have compared ^11^C-acetate and ^11^C-choline uptake in 12 PCa patients and concluded that the ability of both radiotracers to detect known BM was identical, although interindividual variation was high [45]. Yu et al. have tested the feasibility of ^11^C-acetate and ^18^F-FDG for the detection and measurement of response to therapy in 8 patients detected with ≥3 BM diagnosed by ^99m^Tc BS. ^11^C-acetate PET detected BM in all 8 patients, whereas ^18^F-FDG PET detected lesions in 6 of the 7 imaged patients [46]. Beheshti et al. have prospectively compared the potential value of ^18^F-fluorocholine and ^18^F-fluoride PET/CT for BM detection in 38 PCa patients [47]. In case of discrepant results, follow-up was used as validator. Overall, 321 lesions were evaluated in this study. Sixteen malignant osteoblastic lesions were negative with both ^18^F-fluorocholine and ^18^F-fluoride PET/CT. The sensitivity, specificity, and accuracy of PET/CT for PCa BM detection was 81%, 93%, and 86% for ^18^F-fluoride and 74% (*P* = 0.12), 99% (*P* = 0.01), and 85% for ^18^F-fluorocholine PET/CT, respectively. In a later study on 70 patients, Beheshti et al. have calculated that the sensitivity, specificity, and accuracy of ^18^F-fluorcholine PET/CT in detecting PCa BM was 79%, 97%, and 84%, respectively [48]. Eschmann et al. have published a small study on 42 patients aiming to compare the diagnostic accuracy of ^11^C-Choline PET/CT and whole-body WB-MRI [49]. After validation by histology, follow-up, or consensus reading, 88/103 detected lesions were considered as malignant including 44 BMs. Sensitivity, specificity, and accuracy for ^11^C-choline PET/CT were 96.6%, 76.5%, and 93.3%, respectively, and for WB-MRI 78.4%, 94.1%, and 81.0%, respectively. Interestingly, however; whole-body MRI was performed without diffusion reading, thus, lowering its performance.

## 3. CT Scanner

CT scanner imaging is central in the diagnosis of musculoskeletal disorders. In bone malignancies, it is not used as a screening test but as a second-line imaging technique to clear-up abnormal BS uptakes remaining unexplained after standard X-ray or to image suspicion of neurological disorders. 

Recently, the development of multidetector spiral technology has reawakened the potential interest of CT for early detection of BMs. In a recent analysis, Groves et al. have investigated the value of this technique in BM assessment [50]. They have compared BS and bone imaging from the cranium vertex to the knee by 16-slices CT in 43 patients with known malignancy. BS detected BM in 14/43 and CT in 13/43 patients. There were, however, several discordances. Based on their preliminary analysis, Groves et al. concluded that CT with its present performance is unlikely to replace BS for BM screening. In addition, the total accumulated radiation dose required by whole spine CT precludes the systematic use of this technique for the determination of therapeutic response in clinical trials.

## 4. Magnetic Resonance Imaging (MRI) of the Skeleton

MRI is highly sensitive for detecting BM in cancer patients [25, 31, 51–53]. Its superiority over BS has been repeatedly demonstrated [54–56]. It has been used as a “*gold standard*” to evaluate PET for detecting BM and more recently to quantify PCa metastases and measure tumour response to therapy [30, 57]. However, the use of MRI in first line is often presented as “not feasible” putting forward its limited availability, costs, or limitations of published series validating the method [30]. 

**Figure 1:**
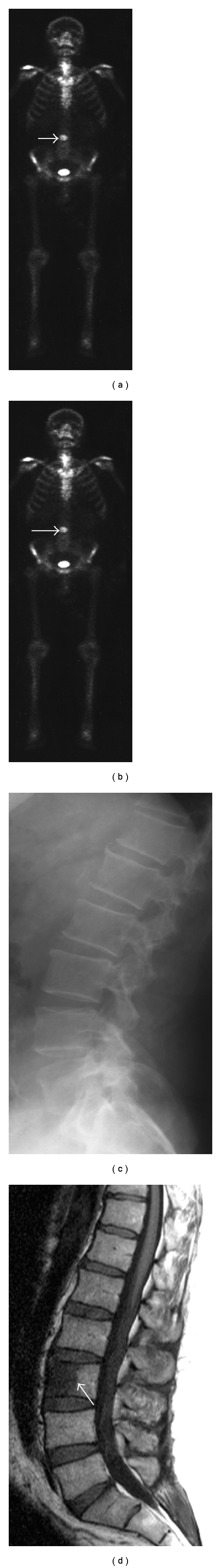
Example of a 55 years old patient diagnosed with high-risk localized disease. ^99m^Tc bone scan shows an area of uptake in the body of the 3rd lumbar vertebra ((a) and (b)). Confirmatory X-ray is read as normal (c) although MRI of the axial skeleton (T1 sequence, (d)) shows a large focal area of malignant replacement of the bone marrow.

MRI characteristics of malignant involvement of the bone marrow are well documented [25, 36, 57–59]. The different MRI patterns of bone marrow involvement in neoplastic disease have been precisely described, so that discriminating metastatic lesions from benign marrow abnormalities such as marrow hyperplasia induced by chemotherapy can easily be made by trained radiologists. MRI appearance of the spinal and pelvi-femoral can be categorized into simple well-defined categories [58, 60]: normal appearance, focal metastatic lesions (focal marrow replacement pattern), which are nodular areas that can be measured, and diffuse marrow infiltration (Figure 1). 

The superiority of MRI lies in its ability to detect early tumour cells seeding into the hematopoietic compartment, leading to replacement of the normal hematopoietic marrow and of its fat cells. This technique, thus, identifies BM at an early stage, before host reaction of the osteoblasts becomes visible on BS and X-ray, therefore, increasing sensitivity [25, 28]. Already in 1993, Turner et al. reported on using MRI in 18 PCa patients to resolve conflicting evidence of BM found on bone scans and X-ray [61]. MRI ruled out metastatic disease in 2 of the 8 positive BS and revealed BM in 5 negative BS. All 5 equivocal BS demonstrated no osseous lesions on MRI. In addition, in 6 patients with evidence of BM serial MRI scans following ADT demonstrated radiographic and clinical improvement. In 2000, Walker et al. evaluated whole-body STIR-MRI to detect metastases to liver, brain, and bone as a single examination in women with breast cancer [62]. BM were identified in 11/17 patients, with correlation between findings at whole-body MRI and scintigraphy in 15/17 patients. Daldrup-Link et al. have compared the diagnostic accuracy of whole-body MRI, Tc-99m BS, and FDG-PET for the detection of bone metastases in 39 children aged 2 to 19 years old with various malignancies [28]. Sensitivities for the detection of bone metastases were 90% for FDG-PET, 82% for MRI, and 71% for skeletal scintigraphy. But FDG-PET also produced most false-positive lesions. More recently, our group has evaluated the diagnostic performance and impact on therapy of one-step MRI of the axial skeleton (MRIas) for detecting BM in 66 patients with high-risk PCa [63]. MRIas has been compared to a routine workup based on BS completed with targeted X-ray in cases of equivocal BS findings and with MRI “on request” (MRIor) in case of inconclusive BS/X-ray findings. Sensitivities were 46% for Tc-99m BS alone, 63% for BS/X-ray, 83% for BS/X-rays/MRI, and 100% for MRIas. Corresponding specificities were 32%, 64%, 100%, and 88%. MRIas was significantly more sensitive than any other approach (*P* < 0.05, McNemar). MRIas correctly identified metastases in 7/23 (30%) patients considered negative, and 8/17 (47%) considered equivocal by other strategies, which resulted in altering the initially planned therapy. This increased sensitivity has clearly an added value in routine practice, both in newly diagnosed PCa, to avoid unnecessary radical therapy, and later on in the disease to enable early initiation of treatment with the hope to prevent or delay the complications of metastases. 

Are these results sufficient to definitively state that MRI will replace BS as initial and sole imaging modality for staging all PCa patients? The answer is no. The access to MRI technology still needs to be improved and the available MRI machine to be increased. Indeed, it is not often acceptable to delay treatment in high-risk patients waiting to rule out BM. In addition, many authors still advocate the use of BS on the assumption that MRI would be too costy [25, 26, 39]. These data, however, demonstrate that MRI surpasses the current imaging strategy used for bone staging in a high-risk patient population. There is place for large multicentric studies in well-defined groups of patients to assess and confirm the clinical efficacy of MRI as the initial tool for bone staging in PCa.

**Figure 2:**
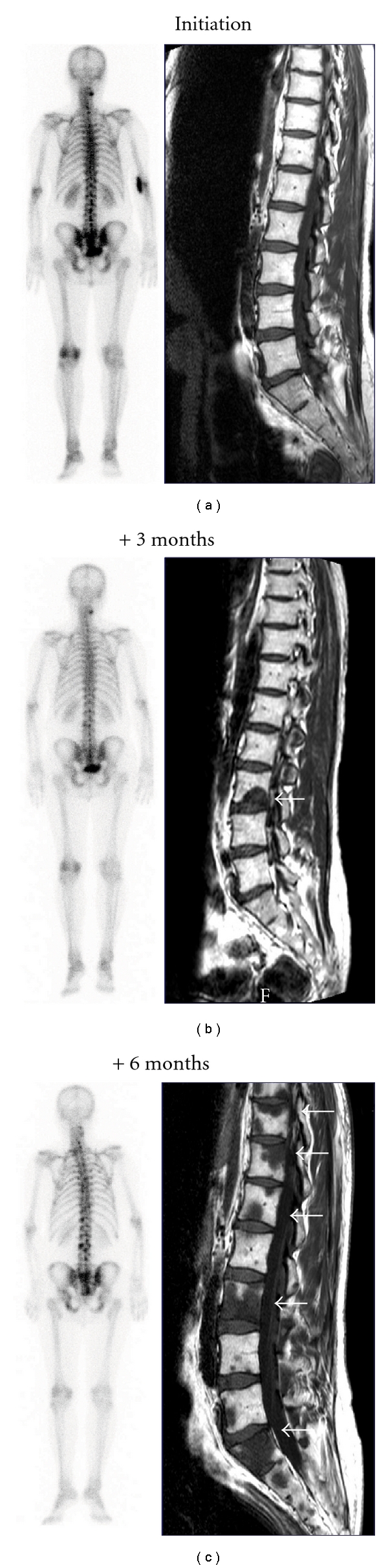
Consecutive series of three ^99m^Tc bone scans (BS) and MRI acquired three months apart on a patient included in a clinical trial designed for M0 CRPC. Only the third BS is adjudicated as positive by the central review although he was already progressive on MRI at the second metastatic workup.

Further development will focus on whole-body MRI (WBMRI). Hardware and software advances have enabled the acquisition of WBMRI, including conventional sequence (T1, STIR) images covering the entire skeleton, and more recently diffusion-weighted images (DWIs), which facilitates the detection of BM, especially in skeletal areas that are difficult to evaluate on conventional sequences, such as ribs [64–68]. In addition, this allows envisioning an all-in-one metastatic workup (visceral and osseous). Eschmann et al. have compared the diagnostic accuracy of ^11^C-Choline PET/CT to whole-body MRI for the staging workup of PCa in 42 patients [44]. Sensitivity, specificity, and accuracy for ^11^C-Choline PET/CT were 96.6%, 76.5%, and 93.3%, respectively, and for MRI 78.4%, 94.1%, and 81.0%. Interestingly, 3 BMs had initially been missed by ^11^C-Choline PET/CT and were found retrospectively. The author concluded that strength of MRI is excellent image quality providing detailed anatomical information whereas the advantage of Choline PET/CT is high image contrast of pathological foci (Figure 2).

## 5. Conclusion

Correct diagnosis of BM has emerged as major challenge for those who are developing new therapeutic strategies, including those who advocate aggressive local treatment of high-risk localized and locally advanced disease. Tc-99m BS may not be over, but the time when patients would be treated on suspicion rather than on robust evidences is passed. Many technologies including metabolic imaging by PET and MRI are indeed rapidly gaining interest in the everyday management of PCa patients. As it is for modern treatments, the diagnosis strategies will be multidisciplinary by nature and involve crossfertilization between nuclearists, urologists, and radiation oncologists.

## References

[1] Ferlay J, Shin HR, Bray F, Forman D, Mathers C, Parkin DM (2010). Estimates of worldwide burden of cancer in 2008: GLOBOCAN 2008. *International Journal of Cancer*.

[2] Verhagen PCMS, Schröder FH, Collette L, Bangma CH (2010). Does Local treatment of the prostate in advanced and/or lymph node metastatic disease improve efficacy of androgen-deprivation therapy? A systematic review. *European Urology*.

[3] Rigaud J, Tiguert R, Le Normand L (2002). Prognostic value of bone scan in patients with metastatic prostate cancer treated initially with androgen deprivation therapy. *Journal of Urology*.

[4] Soloway MS, Hardeman SW, Hickey D (1988). Stratification of patients with metastatic prostate cancer based on extent of disease on initial bone scan. *Cancer*.

[5] Nørgaard M, Jensen AØ, Jacobsen JB, Cetin K, Fryzek JP, Sørensen HT (2010). Skeletal related events, bone metastasis and survival of prostate cancer: a population based cohort study in Denmark (1999 to 2007). *Journal of Urology*.

[6] Loberg RD, Logothetis CJ, Keller ET, Pienta KJ (2005). Pathogenesis and treatment of prostate cancer bone metastases: targeting the lethal phenotype. *Journal of Clinical Oncology*.

[7] Smith MR, Cook RJ, Coleman R (2007). Predictors of skeletal complications in men with hormone-refractory metastatic prostate cancer. *Urology*.

[8] National Comprehensive Cancer Network (2011). Clinical practice guidelines in oncology. Prostatecancer.

[9] Heidenreich A, Bolla M, Joniau S (2010). Guidelines on prostate cancer. *Urology EAo*.

[10] Scher HI, Halabi S, Tannock I (2008). Design and end points of clinical trials for patients with progressive prostate cancer and castrate levels of testosterone: recommendations of the Prostate Cancer Clinical Trials Working Group. *Journal of Clinical Oncology*.

[11] Therasse P (2002). Measuring the clinical response. What does it mean?. *European Journal of Cancer*.

[12] Therasse P, Arbuck SG, Eisenhauer EA (2000). New guidelines to evaluate the response to treatment in solid tumors. European Organization for Research and Treatment of Cancer, National Cancer Institute of the United States, National Cancer Institute of Canada. *Journal of the National Cancer Institute*.

[13] Aus G, Bergdahl S, Lodding P, Lilja H, Hugosson J (2007). Prostate cancer screening decreases the absolute risk of being
diagnosed with advanced prostate cancer-results from a
prospective, population-based randomized controlled trial. *European Urology*.

[14] Rana A, Karamanis K, Lucas MG, Chisholm GD (1992). Identification of metastatic disease by T category Gleason score and serum PSA level in patients with carcinoma of the prostate. *British Journal of Urology*.

[15] Briganti A, Passoni N, Ferrari M (2010). When to perform bone scan in patients with newly diagnosed
prostate cancer: external validation of the currently available
guidelines and proposal of a novel risk stratification tool. *European Urology*.

[16] Thompson I, Thrasher JB, Aus G (2007). Guideline for the management of clinically localized prostate cancer: 2007 update. *Journal of Urology*.

[17] Gomez P, Manoharan M, Kim SS, Soloway MS (2004). Radionuclide bone scintigraphy in patients with biochemical recurrence after radical prostatectomy: when is it indicated?. *BJU International*.

[18] Pound CR, Partin AW, Eisenberger MA, Chan DW, Pearson JD, Walsh PC (1999). Natural history of progression after PSA elevation following radical prostatectomy. *Journal of the American Medical Association*.

[19] Choueiri TK, Dreicer R, Paciorek A, Carroll PR, Konety B (2008). A model that predicts the probability of positive imaging in
prostate cancer cases with biochemical failure after initial
definitive local therapy. *Journal of Urology*.

[20] Smith MR, Kabbinavar F, Saad F (2005). Natural history of rising serum prostate-specific antigen in men with castrate nonmetastatic prostate cancer. *Journal of Clinical Oncology*.

[21] Smith MR, Cook R, Lee K-A, Nelson JB (2011). Disease and host characteristics as predictors of time to first bone metastasis and death in men with progressive castration-resistant nonmetastatic prostate cancer. *Cancer*.

[22] Condon BR, Buchanan R, Garvie NW (1981). Assessment of progression of secondary bone lesions following cancer of the breast or prostate using serial radionuclide imaging. *British Journal of Radiology*.

[23] Pollen JJ, Gerber K, Ashburn WL, Schmidt JD (1981). Nuclear bone imaging in metastatic cancer of the prostate. *Cancer*.

[24] Einhorn TA, Vigorita VJ, Aaron A (1986). Localization of technetium-99m methylene diphosphonate in bone using microautoradiography. *Journal of Orthopaedic Research*.

[25] Eustace S, Tello R, DeCarvalho V (1997). A comparison of whole-body turboSTIR MR imaging and planar 99mTc-methylene diphosphonate scintigraphy in the examination of patients with suspected skeletal metastases. *American Journal of Roentgenology*.

[26] Hamaoka T, Madewell JE, Podoloff DA, Hortobagyi GN, Ueno NT (2004). Bone imaging in metastatic breast cancer. *Journal of Clinical Oncology*.

[27] Rybak LD, Rosenthal DI (2001). Radiological imaging for the diagnosis of bone metastases. *Quarterly Journal of Nuclear Medicine*.

[28] Daldrup-Link HE, Franzius C, Link TM (2001). Whole-body MR imaging for detection of bone metastases in children and young adults: comparison with skeletal scintigraphy and FDG PET. *American Journal of Roentgenology*.

[29] Jacobson AF, Fogelman I (1998). Bone scanning in clinical oncology: does it have a future?. *European Journal of Nuclear Medicine*.

[30] Schirrmeister H, Guhlmann A, Elsner K (1999). Sensitivity in detecting osseous lesions depends on anatomic localization: planar bone scintigraphy versus 18F PET. *Journal of Nuclear Medicine*.

[31] Gosfield E, Alavi A, Kneeland B (1993). Comparison of radionuclide bone scans and magnetic resonance imaging in detecting spinal metastases. *Journal of Nuclear Medicine*.

[32] McKillop JH, McDougall IR (1980). The role of skeletal scanning in clinical oncology. *British Medical Journal*.

[33] Venkitaraman R, Sohaib A, Cook G (2007). MRI or bone scan or both for staging of prostate cancer?. *Journal of Clinical Oncology*.

[34] Schirrmeister H, Glatting G, Hetzel J (2001). Prospective evaluation of the clinical value of planar bone scans, SPECT, and 18F-labeled NaF PET in newly diagnosed lung cancer. *Journal of Nuclear Medicine*.

[35] Han LJ, Au-Yong TK, Tong WCM, Chu KS, Szeto LT, Wong CP (1998). Comparison of bone single-photon emission tomography and planar imaging in the detection of vertebral metastases in patients with back pain. *European Journal of Nuclear Medicine*.

[36] Even-Sapir E, Metser U, Mishani E, Lievshitz G, Lerman H, Leibovitch I (2006). The detection of bone metastases in patients with high-risk prostate cancer: 99mTc-MDP planar bone scintigraphy, single- and multi-field-of-view SPECT, 18F-fluoride PET, and 18F- Fluoride PET/CT. *Journal of Nuclear Medicine*.

[37] Nozaki T, Yasuda K, Akashi T, Fuse H (2008). Usefulness of single photon emission computed tomography imaging in the detection of lumbar vertebral metastases from prostate cancer. *International Journal of Urology*.

[38] Helyar V, Mohan HK, Barwick T (2010). The added value of multislice SPECT/CT in patients with equivocal bony metastasis from carcinoma of the prostate. *European Journal of Nuclear Medicine and Molecular Imaging*.

[39] Schirrmeister H, Guhlmann A, Kotzerke J (1999). Early detection and accurate description of extent of metastatic bone disease in breast cancer with fluoride ion and positron emission tomography. *Journal of Clinical Oncology*.

[40] Schirrmeister H, Kühn T, Guhlmann A (2001). Fluorine-18 2-deoxy-2-fluoro-D-glucose PET in the preoperative staging of breast cancer: comparison with the standard staging procedures. *European Journal of Nuclear Medicine*.

[41] Uematsu T, Yuen S, Yukisawa S (2005). Comparison of FDG PET and SPECT for detection of bone metastases in breast cancer. *American Journal of Roentgenology*.

[42] Ghanem N, Uhl M, Brink I (2005). Diagnostic value of MRI in comparison to scintigraphy, PET, MS-CT and PET/CT for the detection of metastases of bone. *European Journal of Radiology*.

[43] Cook GJR, Fogelman I (2000). The role of positron emission tomography in the management of bone metastases. *Cancer*.

[44] Eschmann SM, Pfannenberg AC, Rieger A (2007). Comparison of 11C-choline-PET/CT and whole body-MRI for staging of prostate cancer. *NuklearMedizin*.

[45] Kotzerke J, Volkmer BG, Glatting G (2003). Intraindividual comparison of [11C]acetate and [11C]choline PET for detection of metastases of prostate cancer. *NuklearMedizin*.

[46] Yu EY, Muzi M, Hackenbracht JA (2011). C11-acetate and F-18 FDG PET for men with prostate cancer bone metastases: relative findings and response to therapy. *Clinical Nuclear Medicine*.

[47] Beheshti M, Vali R, Waldenberger P (2008). Detection of bone metastases in patients with prostate cancer by 18F fluorocholine and 18F fluoride PET-CT: a comparative study. *European Journal of Nuclear Medicine and Molecular Imaging*.

[48] Beheshti M, Vali R, Waldenberger P (2010). The use of F-18 choline PET in the assessment of bone metastases in prostate cancer: correlation with morphological changes on CT. *Molecular Imaging and Biology*.

[49] Eschmann SM, Pfannenberg AC, Rieger A (2007). Comparison of 11C-choline-PET/CT and whole body-MRI for staging of prostate cancer. *NuklearMedizin*.

[50] Groves AM, Beadsmoore CJ, Cheow HK (2006). Can 16-detector multislice CT exclude skeletal lesions during tumour staging? Implications for the cancer patient. *European Radiology*.

[51] Daffner RH, Lupetin AR, Dash N (1986). MRI in the detection of malignant infiltration of bone marrow. *American Journal of Roentgenology*.

[52] Sanal SM, Flickinger FW, Caudell MJ, Sherry RM (1994). Detection of bone marrow involvement in breast cancer with magnetic resonance imaging. *Journal of Clinical Oncology*.

[53] Traill ZC, Talbot D, Golding S, Gleeson FV (1999). Magnetic resonance imaging versus radionuclide scintigraphy in screening for bone metastases. *Clinical Radiology*.

[54] Frank JA, Ling A, Patronas NJ (1990). Detection of malignant bone tumors: MR imaging vs scintigraphy. *American Journal of Roentgenology*.

[55] Haubold-Reuter BG, Duewell S, Schilcher BR, Marincek B, Schulthess GK (1993). The value of bone scintigraphy, bone marrow scintigraphy and fast spin-echo magnetic resonance imaging in staging of patients with malignant solid tumours: a prospective study. *European Journal of Nuclear Medicine*.

[56] Kattapuram SV, Khurana JS, Scott JA, El-Khoury GY (1990). Negative scintigraphy with positive magnetic resonance imaging in bone metastases. *Skeletal Radiology*.

[57] Tombal B, Rezazadeh A, Therasse P, Van Cangh PJ, Vande Berg B, Lecouvet FE (2005). Magnetic resonance imaging of the axial skeleton enables objective measurement of tumor response on prostate cancer bone metastases. *Prostate*.

[58] Vande Berg BC, Lecouvet FE, Michaux L, Ferrant A, Maldague B, Malghem J (1998). Magnetic resonance imaging of the bone marrow in hematological malignancies. *European Radiology*.

[59] Vande Berg BC, Lecouvet FE, Michaux L (1998). Magnetic resonance imaging of the normal bone marrow. *Skeletal Radiology*.

[60] Vanel D, Bittoun J, Tardivon A (1998). MRI of bone metastases. *European Radiology*.

[61] Turner JW, Hawes DR, Williams RD (1993). Magnetic resonance imaging for detection of prostate cancer metastatic to bone. *Journal of Urology*.

[62] Walker R, Kessar P, Blanchard R (2000). Turbo STIR magnetic resonance imaging as a whole-body screening tool for metastases in patients with breast carcinoma: preliminary clinical experience. *Journal of Magnetic Resonance Imaging*.

[63] Lecouvet FE, Geukens D, Stainier A (2007). Magnetic resonance imaging of the axial skeleton for detecting bone metastases in patients with high-risk prostate cancer: diagnostic and cost-effectiveness and comparison with current detection strategies. *Journal of Clinical Oncology*.

[64] Lecouvet FE, Simon M, Tombal B, Jamart J, Vande Berg BC, Simoni P (2010). Whole-body MRI (WB-MRI) versus axial skeleton MRI (AS-MRI) to detect and measure bone metastases in prostate cancer (PCa). *European Radiology*.

[65] Luboldt W, Küfer R, Blumstein N (2008). Prostate carcinoma: diffusion-weighted imaging as potential alternative to conventional MR and11C-choline PET/CT for detection of bone metastases. *Radiology*.

[66] Venkitaraman R, Cook GJR, Dearnaley DP (2009). Whole-body magnetic resonance imaging in the detection of skeletal metastases in patients with prostate cancer. *Journal of Medical Imaging and Radiation Oncology*.

[67] Kwee TC, Takahara T, Ochiai R, Nievelstein RAJ, Luijten PR (2008). Diffusion-weighted whole-body imaging with background body signal suppression (DWIBS): features and potential applications in oncology. *European Radiology*.

[68] Vilanova JC, Barceló J (2008). Diffusion-weighted whole-body MR screening. *European Journal of Radiology*.

